# Molecular Mechanisms of Resistance to Tumour Anti-Angiogenic Strategies

**DOI:** 10.1155/2010/835680

**Published:** 2010-03-09

**Authors:** Renaud Grépin, Gilles Pagès

**Affiliations:** Institute of Developmental Biology and Cancer UMR 6543, University of Nice Sophia Antipolis, CNRS, Centre Antoine Lacassagne, 06189 Nice, France

## Abstract

Tumour angiogenesis, described by Folkman in the early seventies, is an essential, complex, and dynamic process necessary for the growth of all solid tumours. Among the angiogenic factors secreted by the tumour cells, the Vascular Endothelial Growth Factor (VEGF) is one of the most important. Most types of human cancer cells express elevated levels of this proangiogenic factor and its receptors. New molecules, called anti-angiogenic, are developed to impair VEGF pathway and tumour vasculature. Despite important results, the clinical benefits of anti-VEGF therapy are relatively modest and usually measured in weeks or months. Why following anti-angiogenic therapy do some patients respond transiently and then why does tumour grow again and disease progress and which compensatory mechanisms could explain the anti-angiogenic treatment failure?

## 1. Introduction

Tumour angiogenesis, as described by Folkman in the early seventies and confirmed today [1], is an essential, complex, and dynamic process necessary for the growth of all solid tumours. It stipulates that a tumour cannot grow through a defined volume if it is not vascularized. The cores of solid tumours rapidly undergo hypoxic with low oxygen levels and nutrients deficiency. Tumour cells counteract this process by producing angiogenic factors responsible for growth and migration of quiescent endothelial cells of proximal blood vessels. The consequence is the creation of a new vascular network to supply the tumour with oxygen, nutrients, growth factors, proteolytic enzymes, tumours cells, and dissemination in host [2, 3]. Tumour angiogenesis and hypoxia are considered as hallmarks of solid tumours [4, 5]. Among the angiogenic factors secreted by tumour cells, the Vascular Endothelial Growth Factor (VEGF) is one of the most important. Different stimuli are responsible of its production. Among them hypoxia was one of the first described. During the angiogenic switch, the frail existing balance between pro- and anti-angiogenic factors is broken in favour of proangiogenic factors [6]. Most types of human cancer cells express elevated levels of VEGF. They also express VEGF receptors at their surface including VEGF-R1,2,3, VEGF-R3 participating to lymphangiogenesis [7–11]. All these results have allowed the development of therapeutic tools targeting VEGF (Bevacizumab) [12] or their receptors (Tyrosine kinase inhibitors) [13].

## 2. Examples of Antiangiogenic Treatments Commonly Used in Metastatic Cancers

For example, Bevacizumab (Avastin, Roche), a humanized neutralizing monoclonal antibody against VEGF, is the first anti-angiogenic molecule approved by the FDA in 2004. This antibody, which is becoming an important anti-angiogenic treatment, is commonly used in association with a cytotoxic chemotherapy for late stages of colon cancer, nonsmall-cell lung cancer, breast cancer, glioblastoma, and metastatic renal cell carcinoma (mRCC). Sorafenib tosylate (Nexavar, Bayer) and Sunitinib malate (Sutent, Pfizer), two small tyrosine kinase inhibitors (TKI), target multiple signaling pathways including VEGF and platelet-derived growth factors (PDGFs). Sorafenib and Sunitinib are approved by the FDA to treat mRCC as a single agent. Sunitinib is used in gastrointestinal stromal tumours (GISTs) and Sorafenib is used for treating patients with liver cancer. Every year a variety of new anti-angiogenic molecules are developed with more than 800 clinical trials [14].

Despite important results, the clinical benefits of anti-VEGF therapy are relatively modest and usually measured in weeks or months [15]. In some cases patients do not respond to anti-VEGF treatments. For example, Bevacizumab used as a single agent to treat colorectal and nonsmall-cell lung tumours is inefficient [12, 16]. Recently a phase III study of Bevacizumab plus chemotherapy in early-stage colon cancer did not meet its primary endpoint for lowering the risk of the cancer recurrence compared to chemotherapy alone [17]. Discontinuous TKI treatments (4 weeks on/2 weeks off) in patients with metastatic breast cancer or mRCC can carry the risk of tumour progression during drug-free break periods [18, 19]. Thereby, in a preclinical model, rapid tumour revascularisation has been reported after removal of anti-VEGF therapy [20]. Multiple angiogenesis inhibitors have been therapeutically validated in preclinical cancer models and several in clinical trials. Why following anti-angiogenic therapy do some patients respond transiently and then why does tumour growth again and disease progress? Which compensatory mechanisms could explain the anti-angiogenic treatment resistance?

The fraction of nonresponsive patients included in anti-angiogenic clinical trials such as anti-VEGF antibody treatment or tyrosine kinase inhibitors is significant [13, 15, 21]. In these cases anti-angiogenic treatment does not permet to obtain beneficial effects. Hence, there is no cessation or retardation of the tumour growth or increase of survival. Resistance to anti-angiogenic agents can be the result of intrinsic tumour resistances or acquired resistances. Different mechanisms can explain these resistances including redundant angiogenic factors with upregulation of alternative angiogenic signals, induction of hypoxia, selection of more aggressive tumour cells, recruitment of bone marrow-derived proangiogenic cells and inflammatory cells invasion, modification of vascular pericyte coverage, and vessel cooption.

### 2.1. The Angiogenic Redundancy and Alternative Proangiogenic Pathways

**Figure 1:**
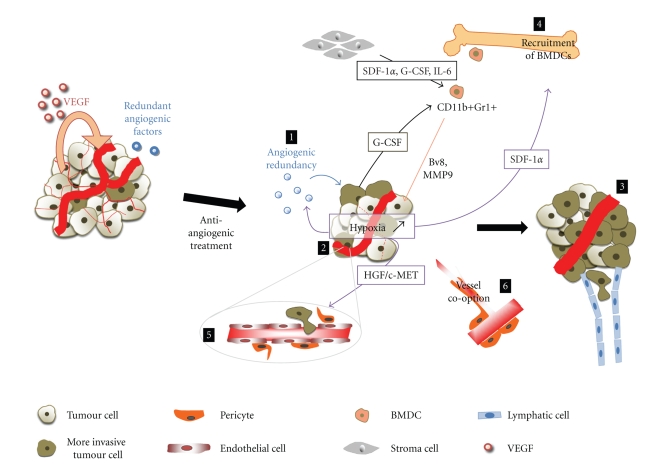
Molecular mechanisms involved in tumor anti-angiogenesis therapies resistance. (1) Following anti-VEGF therapies, redundant angiogenic factors are produced by tumour cells. Antiangiogenic treatments reduce and normalize tumour vasculature but increase intratumour hypoxia (2). Hypoxia induces SDF-1*α* which recruits BMDCs such as CD11b+Gr1+, redundant angiogenic factors (1), and activates HGF/c-MET pathway (5). BMDCs are activated by factors secreted by stroma cells (SDF-1*∝*, G-CSF, and IL-6). Inhibition of VEGF induces endothelial cells apoptosis and pericytes attachment to endothelial cells is loosed (5). New vessels could be recruited to tumour site by vessel cooption (6). Finally mechanisms involved in tumor anti-angiogenesis resistances lead to select more invasive and metastatic tumour cells (3).

The angiogenic redundancy is the first resistance mechanism identified following anti-VEGF therapy [22, 23]. VEGF is the predominant angiogenic factor in human tumours. However, during tumour development redundant proangiogenic factors could be produced including Fibroblast Growth Factors (FGFs), Platelet Derived Growth Factors (PDGFs), Placenta Growth Factor (PlGF), and Tumour Necrosis Factor-*α* (TNF-*α*) [24–26] (Figure 1(1)). For example, in early-stage breast cancers VEGF is the major proangiogenic factor whereas in late-stage additional angiogenic molecules are produced including FGF-2 [23].

Preclinical and clinical anti-VEGF studies have shown that tumours can grow despite VEGF pathway inhibition due to angiogenic redundancy. In a preclinical model of pancreatic neuron-endocrine cancer, after few days of anti-VEGFR2 antibody (DC101) treatment, the vascular density of tumours is reduced and tumours have regions of acute hypoxia. Histological analysis shows that tumours have a wide front of tumour invasion compared to controls and tumours are more invasive after one week of treatment and this increases after four weeks of continuous treatment. Despite the disruption of VEGF angiogenic switch following long-term treatment, a phenotypic resistance to anti-VEGFR2 therapy emerges. During this phase, the tumour's revascularization is increased indicating an active tumour angiogenesis. This progression phase is linked to enhanced production of redundant angiogenic factors, such as FGF family members. If mice are treated with anti-VEGFR, then with FGF-trap just before tumour vasculature regrowth, the tumour growth and neovascularization are attenuated. This independence of VEGF pathway is associated with hypoxia-mediated induction of proangiogenic factors such as FGF-1 and 2, ephrin A1 and 2, and angiopoietin 1 [22]. A synergism between the low expression of two angiogenic factors such as FGF-2 and PDGF-bb could be sufficient to promote angiogenic response although it is expressed at low levels [27, 28]. Following anti-VEGF therapy, levels of plasmatic placental growth factor (PlGF) are increased and seem to be implicated in the angiogenic redundancy [26, 29]. In experimental studies, VEGF-sensitive and resistant tumours respond to PlGF antibody treatment, this antibody enhances the efficiency of anti-VEGFR2 therapy, and it reduces tumour angiogenesis and metastasis without inducing hypoxia [24]. Moreover, anti-PlGF prevents infiltration of angiogenic macrophages. However, clinical studies on VEGF-trap, that binds both VEGF-A and PlGF, do not show an additional benefit compared to Bevacizumab. The withdrawal of one proangiogenic factor could be counterbalanced by production of compensatory angiogenic growth factors and/or chemokines leading to angiogenic rescue program [30]. To prevent angiogenic redundancy different actors implicated in tumour angiogenesis might be targeted at the same time.

### 2.2. Hypoxia: A Major Inducer of Angiogenic Redundancy

Antiangiogenic therapies reduce and normalize tumour vasculature but increase intratumour hypoxia [31, 32] (Figure 1(2)). Hypoxia and overexpression of hypoxia-induced factor-1 (HIF-1) have been associated with radiation therapy and chemotherapy resistance, selection of invasive and metastatic cells, and a poor clinical prognosis of solid tumours [33]. HIF-1 is considered as the major regulator of angiogenic actors following hypoxia. It regulates a lot of genes involved in angiogenesis (VEGF, PlGF, VEGFR-1), proliferation and migration of endothelial cells (such as VEGF, PlGF, FGF2, CXCL12/CXCR4, PDGF), pericytes recruitment (PDGF, Ang-1), and modification of vascular permeability (VEGF/VEGFR-1, Ang-2) [34, 35]. 

The treatment of recurrent glioblastoma patients with a tyrosine kinase inhibitor targeting VEGF receptors initially leads to disease stable but resistances appear after few weeks. The development of resistance following VEGF blockade is associated with an increase of circulating levels of basic FGF, stromal cell-derived factor 1 alpha (SDF1*α*), two genes controlled by HIF-1, and viable circulating endothelial cells [13, 34, 36]. 

Hepatocyte Growth Factor (HGF) is a potent mitogenic, motogenic, morphogenic factor, and also an important actor in angiogenesis and tumour growth [37–39]. HGF induces and activates its membrane tyrosine kinase receptor c-MET. Whereas HGF is mostly produced by mesenchymal cells [40], c-MET is expressed by different cell types such as vascular and lymphatic endothelial cells and pericytes [41]. Thereby, HGF effects are not limited to endothelial cells. HGF binds c-MET and induces its homodimerization and autophosphorylation; then c-MET activates signal transduction pathways such as Src, Akt, MEK, STAT3 [42] and leads to increase expression of VEGF and VEGF/R in endothelial cells [43]. C-MET and HGF are deregulated and correlated with poor prognosis in a lot of human cancers. The receptor can be constitutively phosphorylated, its gene mutated or amplified in tumours. HGF/c-MET promotes cell invasiveness and triggers metastases through angiogenic pathway [44]. It has been described, in lung cancer patient specimens, that HGF is colocalized with fibroblasts. When lung cancer cells and HGF-producing fibroblasts are injected into mice, the tumour becomes resistant to EGFR-TKIs treatment. The stromal fibroblasts seem to an actor in TKI treatment resistance by producing angiogenic factors such as HGF [45]. In a pancreatic cancer model it has been shown that HIF-1 increases c-MET expression in cancer cells and HGF secretion by fibroblast cells [46]. Hypoxia induces HGF/c-MET signaling pathway that leads to matrice membrane degradation and increase of cell migration towards blood or lymphatic vessels (Figure 1(5)). 

The plasma-membrane bound Notch receptor ligand, Delta-like ligand 4 (DLL4), can be an alternative angiogenic pathway which participates to anti-angiogenic treatment failure. DLL4 is highly expressed by vascular endothelial cells and induced by VEGF. It interacts with Notch cell-surface receptors to act as a negative feedback inhibitor downstream of VEGF signaling to restrain the sprouting and branching of new blood vessels [47, 48]. Inhibition of DLL4-Notch signaling induces an increase in vessel density but these blood vessels are abnormal and not perfused. Therefore intratumour hypoxia is increased and leads to induction of transcription of proangiogenic genes regulated by HIF-1 [48–50]. Moreover, tumours that have an intrinsic resistance to anti-VEGF therapy are responsive to inhibition of DLL4/Notch signaling [51].

### 2.3. Selection of More Invasive Tumour Cells

The angiogenic inhibitors normalize tumour vasculature, reduce tumour size, but increase local hypoxia (Figure 1(3)). It has been shown that tumour cells cultured under hypoxia conditions can become more invasive and metastatic [52]. More recently, two studies support the hypothesis that under anti-angiogenic treatment, cancer cells become more invasive and metastatic to migrate to normoxic location. Thereby, in mouse models of pancreatic neuron-endocrine carcinoma and glioblastoma, the primary tumour size decreases after one week of Sunitinib. This anti-angiogenic treatment seems to select more aggressive cancer cells and local tumour cell invasion and distant metastasis are increased. Furthermore, tumours and disseminated liver metastases of animals present more regions of hypoxia compared to untreated control tumours. The proportion of invasive tumours during long-term continuous treatment and the invasive phenotype are not reverse even when the anti-angiogenic treatment is lifted [53]. Another study suggests, by metastasis assays, that a short-term of TKIs treatments reduces tumour growth but increases the incidence of metastasis, facilitates metastatic dissemination of tumour cells, and decreases overall survival of animals. This observation is not reverted when mice are treated one week before tumour cells implantation [54].

### 2.4. Recruitment of Bone Marrow Derived Proangiogenic Cells and Inflammatory Cells Invasion

Antiangiogenic therapies normalize vessels but increase intratumoral hypoxia which lead to recruitment of bone marrow derived cells (BMDCs, endothelial and pericytes progenitors, tumour associated macrophages, immature monocytic cell and myeloid cells) (Figure 1(4)). These cells produce a lot of different proangiogenic factors and can constitute an adaptive mechanism of resistance in low oxygen context. Preclinical and clinical studies have revealed that the number of myeloid cell-derived suppressor cells (MDSCs), such as CD11b+Gr1+ cells, is increased in tumours and peripheral blood of tumour-bearing animals and in blood and spleen of cancer patients [55]. Furthermore the invasion of tumour by these myeloid cells is associated with tumour growth and progression and also contributes to refractoriness to anti-VEGF antibody treatment [56, 57]. Tumours and stromal cells secrete interleukin 6 (IL-6), SDF-1*α*, and granulocyte colony-stimulating factor (G-CSF), three factors implicated in CD11b+Gr1+ cells mobilization and activation [58, 59]. G-CSF expression by tumour or stromal cells is crucial for refractoriness [56]. Moreover, patients treated by tyrosine kinase inhibitors, such as Sorafenib or Sunitinib, or by Bevacizumab plus chemotherapy could be neutropenic [60, 61]. Studies have shown that with a recombinant G-CSF treatment, the use of hematopoietic growth factors for treating patients with neutropenia can mobilize endothelial-cell progenitors and CD11b+Gr1+ [62–64]. 

Furthermore, SDF-1*α* expression by endothelial cells is increased by HIF-1 following hypoxic conditions. BMDCs expressed the SDF-1*α* receptor CXCR4 and are recruited to the ischemic tissue by cell tropism to SDF-1*α*. SDF-1*α* upregulation is associated with anti-angiogenic treatment resistance in patients with glioblastoma [13]. Following Sunitinib treatments, levels of SDF-1*α* and G-CSF are increased dose-dependently in healthy mice and cancer patients [26]. Thereby, in hepatocellular carcinoma, the plasmatic level increase of SDF-1*α* and IL-6, in patients treated with Sunitinib, is associated with a poor outcome [65]. During Bevacizumab therapy, plasmatic levels of SDF-1*α* are increased in patients with rectal cancer and seem to be associated with distant metastasis after three years [66]. Could SDF-1*α* be considered as a biomarker of response or resistance to anti-angiogenic treatment [67]? In response to SDF-1*α* and Lysyl Oxidase gradient, CD11b+Gr1+ cells will be recruited at premetastatic sites and promote tumour metastasis through matrix metalloproteinase 2 (MMP2) production [68, 69]. In the tumour microenvironment and proangiogenic culture conditions, these cells acquire endothelial cell properties [64]. CD11b+Gr1+ cells promote tumour angiogenesis by production of angiogenic factors such as Bv8, a protein related to endocrine-gland derived-VEGF [63] and matrix degrading enzymes like the matrix metalloproteinase 9. Some tumours recruit CD11b+Gr1+ cells from the bone marrow to the tumour site and others do it in response to anti-VEGF treatment [70].

### 2.5. Tumour-Associated Endothelial Cells and Pericytes

Tumour-associated endothelial cells and pericytes were generally assumed to be genetically stable and to have a low mutational rate [71]. Antiangiogenic therapies target endothelial cells which develop less drug resistance than tumour cells. Finally, recent studies have shown that normal and tumour-associated endothelial cells are different and can be tumour type-dependent, aneuploid, and acquire genotypic alterations [72, 73]. Therefore, these endothelial cell alterations in tumour environment can induce a complex crosstalk between angiogenic pathways and increase anti-angiogenic therapies resistances. Endothelial cells secrete PDGF-bb. It mediates proliferation and migration of pericytes which express PDGF-R *β* [74]. Endothelial cells can induce pericyte recruitment in order to be protected from anti-VEGF therapy. This leads to an increase of mature vessels covered with pericytes [75, 76]. Pericytes are vascular smooth muscle lineage cells closely associated with the endothelial cells; this contact enhances endothelial cell survival [77]. These cells are important for vascular development, endothelial cells permeability, vessel stabilization by matrix deposition and/or release, vessel maturation, and remodeling [78]. Pericytes secrete paracrine factors that stimulate signaling pathways implicated in endothelial cell differentiation and survival and then modulating vessel stabilization and maturation [74, 79, 80]. Moreover, in context of tumours or in response to treatments, pericytes express different proteins compared to normal conditions [81]. Anti-VEGF therapies lead to a 80% destruction of the tumour vasculature [82]. Furthermore, vascular sprouting is suppressed, blood flow arrested in some vessels, and finally some tumour vessels regress and others are normalized [32, 83–85]. Inhibition of VEGF pathway may induce endothelial cell apoptosis and/or selection of endothelial cells which express less VEGFR-2 [86] (Figure 1(5)). This reduction of expression is reversible and high reexpression of this receptor corresponds with the return of tumour vessels' dependence for VEGF [20]. VEGFR-2 blockade can lead to the upregulation of angiopoietin 1 that increases pericyte coverage of vessels [87]. Despite the endothelial cells' regression and pericyte changes, the vascular basal membrane of tumours persists and provides a potential scaffold for tumour revascularization and a storage site for angiogenic growth factors [82, 88]. In preclinical study, after 7 days of treatment, endothelial sprouts grew into empty sleeves of basal membrane just one day after anti-VEGF withdrawal. After 7 days, the tumour's regrowth is complete and pericyte phenotypes reverse to return to baseline. Furthermore, tumour vessels become functional, reacquire the dependence to VEGF, and remain sensible to anti-VEGF therapy [20]. Targeting endothelial cells and pericytes by inhibition of VEGF pathway and PDGF receptor with tyrosine kinase inhibitors can increase the efficacy of treatments [89]. Moreover, tumour pericytes have abnormal shapes, lose the attachment to endothelial cells, and contribute to the aberrant tumour vasculature [90]. Contrariwise, the loss of pericyte attachment may disrupt the vascular integrity, increases the risk of hemorrhage, and facilitates the transit and dissemination of tumour cells in circulatory system.

### 2.6. Vessel Cooption

Patients treated by anti-angiogenic therapy are mainly patients with metastases for which other treatments are no longer available. In vasculature-rich organs such as brain, liver, and lung, primary tumours cells and metastases could coopt with the neighboring quiescent normal blood vessels [91] (Figure 1(6)). In rat brain, one or two weeks after C6 glioma cell implantation, small tumours are vascularized without angiogenic response [92]. In early stage of cooption, the tumour growth is angiogenesis independent. Tumours vessels display characteristics of normal vessels. Hence, they will be less sensitive to anti-angiogenic molecules [93, 94].

## 3. Conclusion

The establishment of a new vascular network by angiogenic process is one part of the basis of solid tumour development and dissemination of tumour cells in the organism as metastasis. The core of the tumour rapidly undergoes hypoxic and tumour cells counteract this process by producing angiogenic factors responsible for growth and migration of quiescent endothelial cells of proximal blood vessels. Among the angiogenic factors, the VEGF is one of the most important. Targeting the VEGF/VEGFR pathway represents a major advance in cancer treatments and an important therapeutic option. Despite the transient effect of these expansive anti-VEGF treatments, the enduring clinical responses are rare. Pre-clinical and clinical trials suggest a host response to VEGF inhibition implicated in treatment failure and can participate to progression of secondary disease. Furthermore, the mechanisms of action of anti-angiogenic drugs and resistances against these molecules vary according to the tumour. Resistance or evasion to anti-angiogenic therapies in preclinical models is more and more reported. For example, anti-angiogenic treatments not only normalize tumour vasculature but also reduce pericyte coverage and increase tumour hypoxia and tumour cells can acquire a more invasive; however the molecular mechanisms are not fully understood. Hence, it is crucial to highlight molecular mechanisms or actors implicated in this phenomenon of resistance in order to anticipate the best responders to the treatment and to improve anti-angiogenic drugs or to develop new agents. Tumours produce multiple factors and animal models suggest that different pathways are activated under or following anti-angiogenic therapies. Hence, targeting complementary pathways implicated in tumour angiogenesis would be more efficient. At last, it is also important to find predictive biological markers of objective response or involved in resistance to anti-angiogenic drugs in order to improve therapy efficacy or to propose alternative anti-angiogenic therapy in case of treatment failure.
